# Plan Enrollment of Medicare-Medicaid Dual Enrollees at Colon Cancer Diagnosis: Insights From SEER-Medicare

**DOI:** 10.1093/geroni/igaf122.1210

**Published:** 2025-12-31

**Authors:** Roshani Dahal, Sayeh Nikpay, Helen Parsons

**Affiliations:** University of Minnesota School of Public Health, Minneapolis, Minnesota, United States; University of Minnesota School of Public Health, Minneapolis, Minnesota, United States; University of Minnesota, Minneapolis, Minnesota, United States

## Abstract

Medicare-Medicaid dual status is associated with poorer quality of cancer care and reduced survival rates. Understanding plan enrollment patterns may provide insight into access to cancer care. Our study examined plan enrollment among Medicare beneficiaries diagnosed with cancer nationally. We created a cohort of colorectal cancer patients aged 66 and older, diagnosed between 2016 and 2019, and enrolled in Medicare Parts A and B in the diagnosis month. We linked Surveillance, Epidemiology and End Results data and Master Beneficiary Summary File. Our cohort included 108,947 Medicare beneficiaries diagnosed with colorectal cancer. Approximately 18% (n = 19,634) were Medicare-Medicaid dual enrollees, with median age at diagnosis of 76 years, 57.2% female, predominantly White (68.1%) followed by Black (18.2%), and mostly residing in metropolitan counties (61.7%). Among these, 71.2% were full-benefit duals and 28.8% were partial-benefit duals. At diagnosis, most duals were enrolled in Traditional Medicare (59% overall; 63.9% full-benefit duals), while others in Medicare Advantage plans (41% overall; 53% partial-benefit duals). Notably, half of colorectal cancers had unknown or missing staging, while the remaining cases were stage II (14.2%), stage III (13.2%), stage IV (12.2%), and stage I (10.7%). Our results indicate that most Medicare-Medicare duals with colorectal cancer are enrolled in Traditional Medicare (known for wide provider networks and limited prior authorization). Future studies should compare access to comprehensive cancer care (including palliative care, supportive therapies, social support, end-of-life care for advanced-stage cancers) between full-benefit and partial-benefit duals in Traditional Medicare versus Medicare Advantage, with attention to missing staging and treatment pathways.

